# A simple, practical and complete *O*-time Algorithm for RNA folding using the Four-Russians Speedup

**DOI:** 10.1186/1748-7188-5-13

**Published:** 2010-01-04

**Authors:** Yelena Frid, Dan Gusfield

**Affiliations:** 1 Department of Computer Science, UC Davis, Davis, California, USA

## Abstract

**Background:**

The problem of computationally predicting the secondary structure (or folding) of RNA molecules was first introduced more than thirty years ago and yet continues to be an area of active research and development. The basic *RNA-folding problem *of finding a maximum cardinality, non-crossing, matching of complimentary nucleotides in an RNA sequence of length *n*, has an *O*(*n*^3^)-time dynamic programming solution that is widely applied. It is known that an *o*(*n*^3^) worst-case time solution is possible, but the published and suggested methods are complex and have not been established to be practical. Significant practical improvements to the original dynamic programming method have been introduced, but they retain the *O*(*n*^3^) worst-case time bound when *n *is the only problem-parameter used in the bound. Surprisingly, the most widely-used, general technique to achieve a worst-case (and often practical) speed up of dynamic programming, the *Four-Russians *technique, has not been previously applied to the RNA-folding problem. This is perhaps due to technical issues in adapting the technique to RNA-folding.

**Results:**

In this paper, we give a simple, complete, and practical Four-Russians algorithm for the basic RNA-folding problem, achieving a worst-case time-bound of *O*(*n*^3^/log(*n*)).

**Conclusions:**

We show that this time-bound can also be obtained for richer nucleotide matching scoring-schemes, and that the method achieves consistent speed-ups in practice. The contribution is both theoretical and practical, since the basic RNA-folding problem is often solved multiple times in the inner-loop of more complex algorithms, and for long RNA molecules in the study of RNA virus genomes.

## Background

The problem of computationally predicting the secondary structure (or folding) of RNA molecules was first introduced more than thirty years ago [1-4], and yet continues to be an area of active research and development, particularly due to the recent discovery of a wide variety of new types of RNA molecules and their biological importance. Additional interest in the problem comes from synthetic biology where modified RNA molecules are designed, and from the study of the complete genomes of RNA viruses (which can be up to 11, 000 basepairs in length).

The basic *RNA-folding problem *of finding a maximum cardinality, non-crossing, matching of complementary nucleotides in an RNA sequence of length *n*, is at the heart of almost all methods to computationally predict RNA secondary structure, including more complex methods that incorporate more realistic folding models, such as allowing some crossovers (pseudoknots). Correspondingly, the basic *O*(*n*^3^)-time *dynamic-programming *solution to the RNA-folding problem remains a central tool in methods to predict RNA structure, and has been widely exposed in books and surveys on RNA folding, computational biology, and computer algorithms. Since the time of the introduction of the *O*(*n*^3^) dynamic-programming solution to the basic RNA-folding problem, there have been several practical heuristic speedups [5,6]; and a complex, worst-case speedup of *O*(*n*^3^(*loglogn*)/(*logn*)^1/2^) time [7] whose practicality is unlikely and unestablished. In [5], Backofen et al. present a compelling, practical reduction in space and time using the observations of [6] that yields a worst-case improvement when additional problem parameters are included in the time-bound i.e. *O*(*nZ*) where *n *≤ *Z *≤ *n*^2^. The method however retains an O(*n*^3^) time-bound when only the length parameter *n *is used. Backofen et al. [5] also comment that the general approach in [7] can be sped up by combining a newer paper on the all-pairs shortest path problem [8]. That approach, if correct, would achieve a worst-case bound of (*O *) which is below the *O*(*n*^3^/log *n*) bound established here. But that combined approach is highly complex, uses word tricks, is not fully exposed, and has practicality that is unestablished.

Surprisingly, the most widely-used and known, general technique to achieve a worst-case (and often practical) speed up of dynamic programming, the Four-Russians technique, has not been previously applied to the RNA-folding problem, although the general Four-Russians technique has been cited in some RNA folding papers. There are two possible reasons for this. The first reason is that a widely exposed version of the original dynamic-programming algorithm does not lend itself to application of the Four-Russians technique. The second reason is that unlike other applications of the Four-Russians technique, in RNA folding, it does not seem possible to separate the preprocessing and the computation phases of the Four-Russians method; rather, those two phases are interleaved in our solution.

In this paper, we give a simple, complete and practical Four-Russians algorithm for the basic RNA-folding problem, achieving a worst-case time reduction from *O*(*n*^3^) to *O*(*n*^3^/*log*(*n*)). We show that this time-bound can also be obtained for richer nucleotide matching scoring-schemes, and that the method achieves significant speed-ups in practice. The contribution is both theoretical and practical, since the basic RNA-folding problem is often solved multiple times in the inner-loop of more complex algorithms and for long RNA molecules in the study of RNA virus genomes.

Some of technical insights we use to make the Four-Russians technique work in the RNA-folding dynamic program come from the paper of Graham et. al. [9] which gives a Four-Russians solution to the problem of Context-Free Language recognition. We note that although it is well-known how to reduce the problem of RNA folding to the problem of *stochastic *context-free parsing [10], there is no known reduction to non-stochastic context-free parsing, and so it is not possible to achieve the *O*(*n*^3^/log *n*) result by simply reducing RNA folding to context-free parsing and then applying the Four-Russians method from [9].

### A Formal Definition of the basic RNA-folding problem

The input to the basic RNA-folding problem consists of a string *K *of length *n *over the four-letter alphabet {A, U, C, G}, and an optional integer *d*. Each letter in the alphabet represents an RNA *nucleotide*.

Nucleotides *A *and *U *are called *complimentary *as are the nucleotides *C *and *G*. A *matching *consists of a set *M *of *disjoint *pairs of sites in *K*. If pair (*i*, *j*) is in *M*, then the nucleotide at site *i *is said to *match *the nucleotide at site *j*. It is also common to require a fixed minimum distance, *d*, between the two sites in any match. A match is a *permitted match *if the nucleotides at sites *i *and *j *are complimentary, and |*i *- *j*| >*d*. We let d = 1 for the remainder of the paper for simplicity of exposition, but in general, *d *can be any value from 1 to n. A matching *M *is *non-crossing *or *nested *if and only if it does not contain any four sites *i *<*i' *<*j *<*j' *where (*i*, *j*) and (*i'*, *j'*) are matches in *M*. Graphically, if we place the sites in *K *in order on a circle, and draw a straight line between the sites in each pair in *M*, then *M *is non-crossing if and only if no two such straight lines cross. Finally, a *permitted matching M *is a matching that is non-crossing, where each match in *M *is a permitted match. The basic RNA-folding problem is to find a permitted matching of *maximum cardinality*. In a richer variant of the problem, a *integer scoring matrix B *is given in the input to the problem; a match between nucleotides in sites *i *and *j *in *K *is given score *B*(*i*, *j*). The problem then is to find a matching with the largest total score. Often this scoring scheme is simplified to give a constant score for each permitted *A*, *U *match, and a different constant score for each permitted *C*, *G *match.

### The original *O*(*n*^3^) time dynamic programming solution

Let *S*(*i*, *j*) represent the score for the optimal solution that is possible for the subproblem consisting of the sites in *K *between *i *and *j *inclusive (where *j *>*i*).

Then the following recurrences hold:

*S*(*i*, *j*) = max{

*S*(*i *+ 1, *j *- 1) + *B*(*i*, *j*) **rule a**,

*S*(*i*, *j *- 1) **rule b**,

*S*(*i *+ 1, *j*) **rule c**,

*Max*_*i*<*k*<*j*_*S*(*i*, *k*) + *S*(*k *+ 1, *j*) **rule d**

}

Rule *a *covers all matchings that contain a possible (*i*, *j*) match; Rule *b *covers all matchings when site *j *is not in any match; Rule *c *covers all matchings when site *i *is not in any match; Rule *d *covers all matchings that can be decomposed into two non-crossing matchings in the interval *i..k*, and the interval *k *+ 1.. *j*. In the case of Rule *d*, the matching is called a *bipartition*, and the interval *i..k *is called the **head **of bipartition, and the interval *k *+ 1.. *j *is the called the **tail **of the bipartition.

These recurrences can be evaluated in nine different ordering of the variables *i*, *j*, *k *[11]. A common suggestion [10-13] is to evaluate the recurrences in order of increasing distance between *i *and *j*. That is, the solution to the RNA folding problem is found for all substrings of *K *of length two, followed by all substrings of length three, etc. up to length *n*. This dynamic programming solution is widely published in textbooks, and it is easy to establish that it is correct and that it runs in *O*(*n*^3^) worst-case time. However, we have not found it possible to apply the Four-Russians technique using that algorithmic evaluation order, but will instead use a different evaluation order.

### An alternative *O*(*n*^3^)-time dynamic programming solution

   **for ***j *= 2 to *n ***do**

      **[Independent] ***Calculations below don't depend on the current column j*

   **for ***i *= 1 to *j *- 1 **do **{rules a and b}

      S(i, j) = max( S(i+1, j-1)+B(i, j), S(i, j-1))

   **[Dependent] ***Calculations below depend on the current column j*

   **for ***i *= *j *- 1 to 1 **do **

      S(i, j) = max(S(i+1, j) , S(i, j) ) (Rule c)

   **for ***k *= *j *- 1 to i+1 **do **{The loop is called the Rule d loop}

      S(i, j) = max(S(i, j), S(i, k-1)+S(k, j) ) (Rule d)

The recurrences used in this algorithm are the same as before, but the order of evaluation of S(i, j) is different. It is again easy to see that this Dynamic Programming Algorithm is correct and runs in *O*(*n*^3^) worst-case time. We will see that this Dynamic Programming algorithm can be used in a Four-Russians speed up.

## Methods

In the Second Dynamic Programming algorithm, each execution of the loop labeled "independent" takes *O*(*n*) time, and is inside a loop that executes only *O*(*n*) times, so the independent loop takes *O*(*n*^2^) time in total, and does not need any improvement. The cubic-time behavior of the algorithm comes from the fact that there are three nested loops, for *j*, *i *and *k *respectively, each incrementing *O*(*n*) times when entered. The speed-up we will obtain will be due to reducing the work in the Rule d loop. Instead of incrementing *k *through each value from *j *- 1 down to *i *+ 1, we will combine indices into groups of size *q *(to be determined later) so that only constant time per group will be needed. With that speed up, each execution of that Rule d loop will increment only *O*(*n/q*) times when entered. However, we will also need to do some preprocessing, which takes time that increases with *q*. We will see that setting *q *= log_3_(*n*) will yield an *O*(*n*^3^/log *n*) overall worst-case time bound.

### Speeding up the computation of *S*

**Figure 1:**
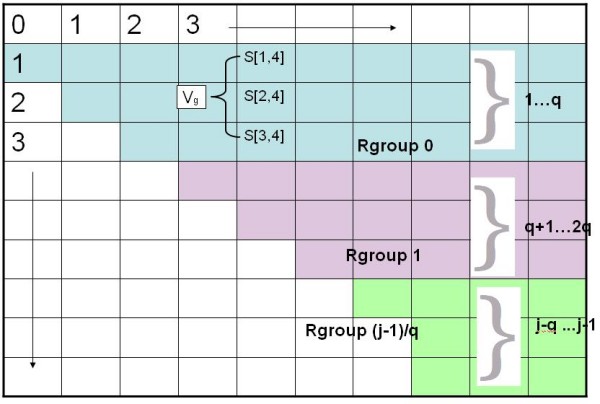
**Rgroup**. Rgroup example with *q *= 3.

We now begin to explain the speed-up idea. For now, assume that *B*(*i*, *j*) = 1 if (*i*, *j*) is a permitted match, and is 0 otherwise. First, conceptually, divide each *column *in the *S *matrix into groups of *q *rows, where *q *will be determined later. For this part of the exposition, suppose *j *- 1 is a multiple of *q*, and let rows 1... *q *be in a group called Rgroup 0, rows *q *+ 1..2*q *be in Rgroup 1 etc, so that rows *j - q...j *- 1 are Rgroup ⌊*j *- 1⌋/*q*. We use *g *as the index for the groups, so *g *ranges from 0 to (⌊*j *- 1⌋/*q*). See Figure 1. We will modify the Second Dynamic Program so that for each fixed *i*, *j *pair, we do not compute Rule d for each *k *= *j *- 1 down to *i *+ 1. Rather, we will only do constant-time work for each Rgroup g that falls completely into the interval of rows from *j *- 1 down to *i *+ 1. For the (at most) one Rgroup that falls partially in that interval, we execute the Rule d loop as before. Over the entire algorithm, the time for those partial intervals is *O*(*n*^2^*q*), and so this detail will be ignored until the discussion of the time analysis.

#### Introducing vector *V*_*g *_and modified Rule d loop

We first modify the Second Dynamic Program to accumulate auxiliary vectors *V*_*g *_inside the Rule d loop. For a fixed *j*, consider an Rgroup *g *consisting of rows *z*, *z *- 1, *z *- *q *+ 1, for some *z *<*j*, and consider the associated consecutive values *S*(*z*, *j*), *S*(*z *- 1, *j*)... *S*(*z *- *q *+ 1, *j*). Let *V*_*g *_be the vector of *those *values in that order.

The work to accumulate *V*_*g *_may seem wasted, but we will see shortly how *V*_*g *_is used.

#### Introducing *v*_*g*_

It is clear that for the simple scoring scheme of *B*(*i*, *j*) = 1 when (*i*, *j*) is a permitted match, and *B*(*i*, *j*) = 0 when (*i*, *j*) is not permitted, *S*(*z *- 1, *j*) is either equal to S(z, j) or is one more then S(z, j). This observation holds for each consecutive pair of values in *V*_*g*_. So for a single Rgroup *g *in column *j*, the change in consecutive values of *V*_*g *_can be encoded by a vector of length *q *- 1, whose values are either 0 or 1. We call that vector *v*_*g*_. We therefore define the function *encode *:*V*_*g *_→ *v*_*g*_such that *v*_*g*_[i] = *V*_*g*_[i-1]-*V*_*g*_[i]. Moreover, for any fixed *j*, immediately after all the *S *values have been computed for the cells in an Rgroup *g*, function *encode*(*V*_*g*_) can be computed and stored in *O*(*q*) time, and *v*_*g *_will then be available in the Rule d loop for all *i *smaller than the smallest row in *g*. Note that for any fixed *j*, the time needed to compute all the encode functions is just *O*(*n*).

#### Introducing Table *R *and the use of *v*_*g*_

We examine the action of the Second Dynamic-Programming algorithm in the Rule d loop, for fixed *j *and fixed *i *<*j *- *q*. For an Rgroup *g *in column *j*, let k*(*i*, *g*, *j*) be the index *k *in Rgroup *g *such that *S*(*i*, *k *- 1) + *S*(*k*, *j*) is maximized, and let S*(*i*, *g*, *j*) denote the actual value *S*(*i*, k*(*i*, *g*, *j*) - 1) + *S*(k*(*i*, *g*, *j*), *j*).

Note that the Second Dynamic-Program can find k*(*i*, *g*, *j*) and S*(*i*, *g*, *j*) during the execution of the Rule *d *loop, but would take *O*(*q*) time to do so. However, by previously doing some preprocessing (to be described below) before column *j *is reached, we will reduce the work in each Rgroup *g *to *O*(1) time. To explain the idea, suppose that before column *j *is reached, we have *precomputed *a table *R *which is indexed by *i*, *g*, *v*_*g*_. Table *R *will have the property that, for fixed *j *and *i *<*j *- *q*, a *single lookup *of *R*(*i*, *g*, *v*_*g*_)) will effectively return k*(*i*, *g*, *j*) for any *g*. Since *k *- 1 <*j *and *k *>*i*, both values *S*(*i*, k*(*i*, *g*, *j*) - 1) and *S*(k*(*i*, *g*, *j*), *j*) are known when we are trying to evaluate *S*(*i*, *j*), so we can find *S*(*i*, k*(*i*, *g*, *j*) - 1) + *S*(k*(*i*, *g*, *j*), *j*) in *O*(1) operations once k*(*i*, *g*, *v*_*g*_) is known.

Since there are *O*(*j/q*) Rgroups, it follows that for fixed *j *and *i*, by calling table *R *once for each Rgroup, only *O*(*j/q*) work is needed in the Rule d loop. Hence, for any fixed *j*, letting *i *vary over its complete range, the work will be *O*(*j*^2^/*q*), and so the total work (over the entire algorithm) in the Rule d loop will be *O*(*n*^3^/*q*). Note that as long as *q *<*n*, some work has been saved, and the amount of saved work increases with increasing *q*. This use of the R table in the Rule d loop is summarized as follows:

### Dependent section using table *R*

   **for ***g *= ⌊(*j *- 1)/*q*⌋ to ⌊(*i *+ 1)/*q*⌋ **do**

      retrieve *v*_*g *_given *g*

      retrieve k*(*i*, *g*, *j*) *from R*(*i*, *g*, *v*_*g*_)

      S(i, j) = max( S(i, j),

      *S*(i, k*(*i*, *g*, *j*) - 1) + *S*(k*(*i*, *g*, *j*), *j*) );

Of course, we still need to explain how *R *is precomputed.

### Obtaining table *R*

Before explaining exactly where and how table *R *is computed, consider the action of the Second Dynamic-Programming algorithm in the Rule d loop, for a fixed *j*. Let *g *be an Rgroup consisting of rows *z *- *q *+ 1, *z *- *q*,..., *z*, for some *z *<*j*. A key observation is that if one knows the single value *S*(*z*, *j*) and the entire vector *v*_*g*_, then one can determine all the values *S*(*z *- *q *+ 1, *j*)... *S*(*z*, *j*) or *V*_*g*_. Each such value is exactly *S*(*z*, *j*) plus a partial sum of the values in *v*_*g*_. In more detail, for any *k *∈ *g*, . Let *decode *(*v*_*g*_) be a function that returns the vector *V' *where .

Next, observe that if one does *not *know any of the *V*_*g *_in the rows of *g *(e.g., the values *S*(*z *- *q *+ 1, *j*), *S*(*z *- 1, *j*)... *S*(*z*, *j*)), but *does *know all of *v*_*g*_, then, for any fixed *i *below the lowest row in Rgroup *g *(i.e., row z-q+1), one can find the value of index *k *in Rgroup *g *to maximize *S*(*i*, *k *- 1) + *S*(*k*, *j*). That value of *k *is what we previously defined as k*(*i*, *g*, *j*). To verify that k*(*i*, *g*, *j*) can be determined from *v*_*g*_, but without knowing any *S *values in column *j*, recall that since *k *- 1 <*j*, *S*(*i*, *k *- 1) is already known. We call this Fact 1.

### Precomputing the *R *table

We now describe the preprocessing that is needed to compute table *R*.

**Figure 2:**
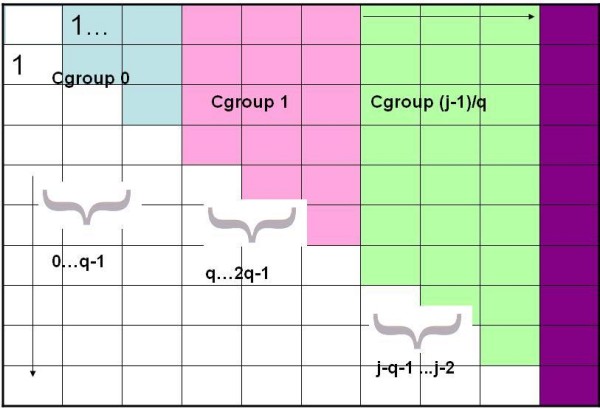
**Cgroup**. Cgroup example.

Conceptually divide matrix for *S *into groups of *columns *of size *q*, i.e., the same size groups that divide each column. Columns 1 through *q *- 1 are in a group we call Cgroup 0, *q *through 2*q *- 1 are in Cgroup 1 etc, and we again use *g *to index these groups. Figure 2 illustrates the division of columns into groups of size *q*. Assume we run the Second Dynamic Program until *j *reaches *q *- 1. That means that all the *S*(*i*, *j*) values have been completely and correctly computed for all columns in Cgroup 0. At that point, we compute the following:

   **for **each binary vector *v *of length *q *- 1 **do**

      *V' *= *decode(v)*

      **for **each *i *such that *i *<*q *- 1 **do**

         *R*(*i*, 0, *v*) is set to the index *k *in Rgroup 0 such that S(i, k-1) + *V'*[k] is maximized. {we let k* denote that optimal *k *}

The above details the preprocessing done after all the *S *values in Cgroup 0 have been computed. In general, for Cgroup *g *> 0, we could do a similar preprocessing after all the entries in columns of Cgroup g have been computed. That is, k*(i, g, v) could be found and stored in R(i, g, v) for all *i *<g * *q*.

This describes the preprocessing that is done after the computation of the *S *values in each Rgroup *g*. With that preprocessing, the table *R *is available for use when computing *S *values in any column *j *>*g *× *q*. Note that the preprocessing computations of table *R *are interleaved with the use of table *R*. This is different than other applications of the Four-Russians technique that we know of. Note also that the amount of preprocessing work increases with increasing *q*. Several additional optimizations are possible, of which one, parallel computation, is described in Results and Discussion section.

With this, the description of the Four-Russians speed up is complete. However, as in most applications of the Four-Russians idea, *q *must be chosen carefully. If not chosen correctly, it would seem that the time needed for preprocessing would be greater than any saving obtained later in the use of *R*. We will see in the next section that by choosing *q *= *log*_3_(*n*) the overall time bound is *O*(*n*^3^/log(*n*)).

### Pseudo-code for RNA folding algorithm with Four Russians SpeedUp

   **for ***j *= 2 to *n ***do **

      **[Independent] ***Calculations below don't depend on the current column j*

      **for ***i *= 1 to *j *- 1 **do **

         S(i, j) = max( S(i+1, j-1)+B(i, j) , S(i, j-1)) (Rules a, b )

      **[Dependent] ***Calculations below depend on the current column j*

      **for ***i *= *j *- 1 to 1 **do **

         **for ***g *= ⌊(*j *- 1)/*q*⌋ to ⌊(*i *+ 1)/*q*⌋ **do **

            **if **(*i *>*k*), *k *∈ Rgroup *g*. **then **{this statement runs at most once, for the smallest *g*}

               find k*(*i*, *g*, *j*) by directly computing and comparing S(i, k-1)+S(k, j) where *k *∈ L = {g * *q *to *i*}

               |*L*| <*q*

            **else **

               retrieve *v*_*g *_given *g*

               retrieve k*(*i*, *g*, *j*) *from R*(*i*, *g*, *v*_*g*_)

               S(i, j) = max( S(i, j), *S*(*i*, k*(*i*, *g*, *j*) - 1) + *S*(k*(*i*, *g*, *j*), *j*) );

         **if **((*i *- 1) mod *q *== 0), Compute *v*_*g *_for group g and store it

      **[Table] **once Cgroup *g *= ⌊*j/q*⌋ is complete

      **for **each binary vector *v *of length *q *- 1 **do**

         *V' *= *decode(v)*

         **for **each *i *1 to *i *<*j *- 1 **do**

            *R*(*i*, *g*, *v*) is set to the index *k *in Rgroup g such that

            S(i, k-1) + *V'*[k] is maximized.

## Results and Discussion

### Correctness and Time analysis

From Fact 1, it follows that when the algorithm is in the Rule d loop, for some fixed *j*, and needs to use *R *to look up k*(*i*, *g*, *j*) for some *g*, k*(*i*, *g*, *j*) = *R*(*i*, *g*, *v*_*g*_). It follows immediately that the algorithm does correctly compute all of the *S *values, and fills in the complete *S *table. A standard traceback can be done to extract the optimal matching, in *O*(*n*) time if pointers were kept when the *S *table built.

#### Time Analysis

The time analysis for any column *j *can be broken down into the sum of the time analyzes for the **[independent]**, **[dependent]**, **[table] **sections.

For any column *j *the **[Independent] **section of the speedup algorithm remains unchanged from the original algorithm and is *O*(*n*) time.

For each row *i*, the **[Dependent] **section of the speedup algorithm is now broken down into *n/q *calls to the table *R*. As discussed above, the total time to compute all the encode functions is *O*(*n*) per column, and this is true for all decode functions as well. Therefore in any column *j*, the dependent section takes *O* time. Also, there is an additional overhead of processing of the one (possible) partial group in any column *j *only takes *O*(*nq*) time. Reversing the order of evaluation in the algorithm for *i *and *k *would eliminate this overhead. However, for simplicity of exposition we leave those details out.

The **[Table] **section sets R(i, g, v) by computing every binary vector *v *and then computing and storing the value k*(i, g, v). The variable *i *ranges from 1 to n and there are 2*^q-^*^1 ^binary vectors. Hence the table section for any complete group *g *takes *O*(q * n * 2*^q-^*^1^) time. There are *n/q *total groups possible.

In summary, the total time for the algorithm is *O*(*n*^2 ^* q) + *O* + *O*(*n*^2^* 2*^q^*) time.

**Theorem 1**. If 2 <*b *<*n*, then the algorithm runs in *O*(*n*^3^/log_*b*_(*n*)) time.

*Proof *Clearly, *O* = *O*(*n*^3^/log_*b*_(*n*)) and *O*(*n*^2 ^log_*b*_(*n*) = *O*(*n*^3^/log_*b*_(*n*)), so we concentrate on the third term in the time bound. To show that *n*^2 ^*× * = *O*(*n*^3^/log_*b*_(*n*)) for 2 <*b *<*n*, we need to show that

The relation holds if

for *z *= log_*b*_(2). 0 <*z *< 1 since *b *> 2.

The above relation holds if

We simplify the above equation to 

We find the limit by taking the derivative of top and bottom (L' Hopital's Rule)

### Parallel Computing

By exploiting the parallel nature of the computation for a specific column *j*, one can achieve an overall time bound of *O*(q * *n*^2^) with vector computing.

The [Independent] section computes max( S(i+1, j-1)+B(i, j), S(i, j-1)), and all three of the values are available for all *i *simultaneously. As a result, it is possible to compute the maximum in parallel, for each *i*, with an asymptotic run time of O(1).

Let's examine the [Dependent] section at a particular *i *and *g*.

It is important to note that when Rgroup *g *is not complete, in other words *i *is greater than *k *(where *k *∈ *Rgroup g*), the maximum fold cannot be found in parallel. The asymptotic time for the *if *branch remains the same as in the non-parallel algorithm: O(n*q) comparisons for a particular column. As stated before, reversing the order of evaluation in the algorithm for *i *and *k *would eliminate this overhead. During the execution of the *else *branch (starting with instruction retrieve k*(*i*, *g*, *j*) *from R*(*i*, *g*, *v*_*g*_) ) there are three observations that lead to a parallel algorithm.

First, observe that *i *is smaller than all the values in Rgroup *g*. This observation holds true because the *else *branch is only taken in that case. Second, because *i *is smaller than any *k *∈ Rgroup *g*, then *v*_*g *_can be computed from the values Rgroup *g *of column j. Third, observe that *R*(*i'*, *g*, *v*_*g*_) is set to k*(*i'*, *g*_*v*_, *j*) for all *i' *<*i *for the particular *v*_*g*_.

We can simultaneously for all *i' *<*i *run:

   retrieve k*(*i'*, *g*, *j*) *from R*(*i'*, *g*, *v*_*g*_)

   *S*(*i'*, *j*) = *max*(*S*(*i'*, *j*), *S*(*i'*, k*(*i'*, *g*, *j*) - 1) + *S*(k*(*i'*, *g*, *j*), *j*) );

Therefore instead of sequentially examining group *g *for each *i' *(such that *i' *<*i*), the parallel algorithm examines group *g *simultaneously. When executed in parallel, the *else *branch computes in *O*(1) time all k* belonging to g for all *i'*. For each *i' *there is at most *n/q *entries in the R table - one for every group. As a result the total time spent in column *j *for the *else *branch is *O*(*n/q*) for all *i *rows. In addition, each column gets encoded into vectors *v*_*g *_of size *q *in *O*(*n*) time. In total, computing in parallel the maximum folding scores for subsequences starting at every possible position *i *and ending at position *j *takes *O*(q * n + *n/q*) = *O*(q * n) time.

The [Table] section can also be done in parallel to find k* for all possible v vectors in O(1) time, entering all 2^*q*-1 ^values into table R simultaneously. The entire algorithm then takes *O*(q * *n*^2^) time in parallel. As stated previously, reordering *i *and *k *would remove the overhead lowering the asymptotic time to *O*(*n*^2^).

### Generalized scoring schemes for B(i, j)

The Four Russians Speed-Up could be extended to any B(i, j) for which all possible differences between S(i, j) and S(i+1, j) do not depend on *n*. Let *C *denote the size of the set of all possible differences. The condition that C doesn't depend on *n *allows one to incorporate not just pairing energy information but also energy information that is dependent on the distance between matching pairs and types of loops. In fact the tabling idea currently can be applied to any scoring scheme as long as the marginal score (*S*(*i *- 1, *j*) - *S*(*i*, *j*)) is not Ω(*n*). In this case, the algorithm takes *O*(n * (n * *C*^*q*-1 ^+  + *n*)) = *O*(*n*^2^*C*^*q*-1 ^+  + *n*^2^) time. If we let *q *= log_*b*_(*n*) with *b *>*C*, the asymptotic time is again *O*(*n*^3^/*log*(*n*)). Based on the proof of Theorem 1, the base of the log must be greater then *C *in order to achieve the speed up. The scoring schemes in [14,15] have marginal scores that are not dependent on *n*, so the speedup method can be applied in those cases.

### Empirical Results

We compare our Four-Russians algorithm to the original *O*(*n*^3^)-time algorithm. The empirical results shown in Table 1 give the average time for 50 tests of randomly generated RNA sequences and 25 downloaded sequences from GenBank, for each size between 1, 000 bp and 6, 000 bp. GenBank sequences varied in actual length and were not exactly the length of the simulated data. However they differed by no more than 30 bp. The algorithm performs identically for randomly generated and GenBank sequences of equal length. This is to be expected because the algorithm's run time is sequence character independent. For all tests where sequences are of the same length, run-times have a standard deviation of at most .01 seconds.

**Table 1:** Computation time (seconds)

*Size*	*O*(*n*^3^) Algorithm	*O*(*n*^3^/log(*n*)) Algorithm	ratio
1000	3.20	1.43	2.23
2000	27.10	7.62	3.55
3000	95.49	26.90	3.55
4000	241.45	55.11	4.38
5000	470.16	97.55	4.82
6000	822.79	157.16	5.24

The purpose of these empirical results is to show that despite the additional overhead required for the Four-Russians approach, it does provide a consistent practical speed-up over the *O*(*n*^3^)-time method, and does not just yield a theoretical result. In order to make this point, we keep all conditions for the comparisons the same, we emphasize the ratio of the running times rather than the absolute times, and we do not incorporate any additional heuristic ideas or optimizations that might be appropriate for one method but not the other. However, we are aware that there are speedup ideas for RNA folding, which do not reduce the *O*(*n*^3^) bound, but provide significant practical speedups. Our empirical results are not intended to compete with those results, or to provide a finished competitive *program*, but to illustrate the practicality of the Four-Russians method, in addition to its theoretical consequences. In future work, we will incorporate all known heuristic speedups, along with the Four-Russians approach, in order to obtain an RNA folding *program *which can be directly compared to all existing methods.

## Conclusions

Extending the Four-Russians speedup by interleaving preprocessing with computation can lead to a practical and simple *O*(*n*^3^/*logn*) time RNA folding algorithm. Through further analysis this basic algorithm could be applied to a variety of scoring schemes, and energy functions. In parallel the speedup can improve the algorithm to run in *O*(*n*^2^) time.

## Competing interests

The authors declare that they have no competing interests.

## Authors' contributions

YF and DG jointly contributed to the conception, design, analysis and interpretation of the algorithm, and jointly contributed to the writing and editing of the manuscript. Implementaton and testing was done by YF.

## Acknowledgements

This research was partially supported by NSF grants SEI-BIO 0513910, CCF-0515378, and IIS-0803564.
